# Survival Nomogram for Metastasis Colon Cancer Patients Based on SEER Database

**DOI:** 10.3389/fgene.2022.832060

**Published:** 2022-02-09

**Authors:** Qinwen Tai, Wei Xue, Mengying Li, Shuli Zhuo, Heng Zhang, Fa Fang, Jinhui Zhang

**Affiliations:** ^1^ Department of General Surgery, Shenzhen Hospital, Southern Medical University, Shenzhen, China; ^2^ Department of Pharmacy, Ruikang Hospital Affiliated to Guangxi University of Chinese Medicine, Nanning, China; ^3^ The First College of Clinical Science, Anhui Medical University, Hefei, China; ^4^ Medical College of Shaoguan University, Shaoguan, China

**Keywords:** metastasis colon cancer, prognosis, SEER, nomogram, treatment

## Abstract

**Introduction:** A prediction model for the 1-, 3-, and 5-year survival rates of metastatic colon cancer (mCC) patients was developed by analyzing important risk factors for the prognosis of mCC patients based on the SEER database.

**Method:** The characteristic of 10,946 patients diagnosed with mCC between 2010 and 2015 was obtained from the SEER database. The population was randomly divided into a training cohort and an internal validation cohort in a 7:3 ratio. Univariate and multivariate cox for independent predictors of mCC prognosis were performed, and nomogram was constructed. The accuracy of the model was verified by calibration curves, ROC curves, and C-index, and the clinical utility of the model was analyzed using decision analysis curves.

**Result:** Age, primary site, grade, surgery, and other eight factors were significantly associated with the prognosis of mCC patients, and these predictors were included in the construction of the nomogram. The C-index was 0.731 (95% CI 0.725–0.737) and 0.736 (95% CI 0.726–0.746) for the training cohort and the validation set, respectively. The results of the ROC curve analysis indicated that the area under the curve (AUC) exceeded 0.7 for both the training cohort and the validation set at 1, 3, and 5 years.

**Conclusion:** The constructed prediction model had an excellent predictive accuracy, which will help clinical decision-making of mCC patients after surgery and individualized treatment.

## Introduction

Colon cancer (CC), an aggressive malignant tumor, ranks 5th in terms of incidence and 5th on the list of cancer-related mortality among all cancers (Sung et al., 2021). In 2020, More than 1.1 million new cases are diagnosed with CC and appropriately 570,000 deaths were attributed to CC (Sung et al., 2021). Over the past few decades, the mortality rate of colon cancer has been on the rise due to a general improvement in living standards and changes in lifestyle (Sung et al., 2021). With the widespread availability of early screening, improving treatment options (including immunotherapy, surgical resection, chemotherapy and radiotherapy), and the discovery of new biomarkers, the prognosis of CC patients has improved significantly (Lemini et al., 2019; Sucandy et al., 2020; Guevara-Cuellar et al., 2021). However, given that the 5-year survival rate (OS) of CC is relatively high, more and more researches have investigated the influencing factors for CC prognosis. In general, the factors that affect the prognosis of CC mainly come from the tumor aspect, including the size of the tumor, the location of the tumor, and the depth of tumor infiltration (Feng et al., 2021; Huang et al., 2021; Wang et al., 2021). Of course, distant metastasis also contributed to the reduction in the survival of CC patients (Chang et al., 2017; Nakamura et al., 2021). Clinically, CC can easily metastasize to other parts of the body, the most frequently metastatic organs were lung and liver, followed by bone and brain (Wang et al., 2020). Metastatic colon cancer (mCC) has been considered a systemic disease, more than 65% of patients with advanced CC recur after surgical treatment (van der Stok et al., 2017), and most patients also experience disease progression due to resistance to targeted and chemotherapeutic agents (Skarkova et al., 2019). Therefore, mCC patients, as a special group, deserve further study.

The tumor lymph node metastasis (TNM) staging system, proposed by the International Union Against Cancer (UICC) and the American Joint Committee on Cancer (AJCC), is the standard method for staging malignant tumors and is extensively used to assess the prognosis of cancer patients (Hari et al., 2013). Of note, TNM staging system has an inherent limitation, as it assesses the risk of individual patients by only three variables (T stage, N stage and M stage) and cannot be combined with other clinical and pathological characteristics like age, sex, ethnicity, tumor size, which has been recognized as risk factors for cancers (Duijster et al., 2021; Neazy et al., 2021). Therefore, nomogram has emerged as a more advanced method owing to its ability to estimate individualized risk based on more comprehensive disease and patient characteristics (Valentini et al., 2011; Balachandran et al., 2015).

In this study, after obtaining clinical data from the SEER database, we grouped the patients in a ratio of 7:3. Of these, 70% were used to build a model by retrospectively analyzing the data to find prognostic factors, and then to construct a predictive nomogram to initially assess the overall survival (OS) of mCC patients at 1, 3 and 5 years. The remaining 30% was used as an internal validation cohort to verify the validity of the nomogram.

## Materials and Methods

### Patients Selection

**FIGURE 1:**
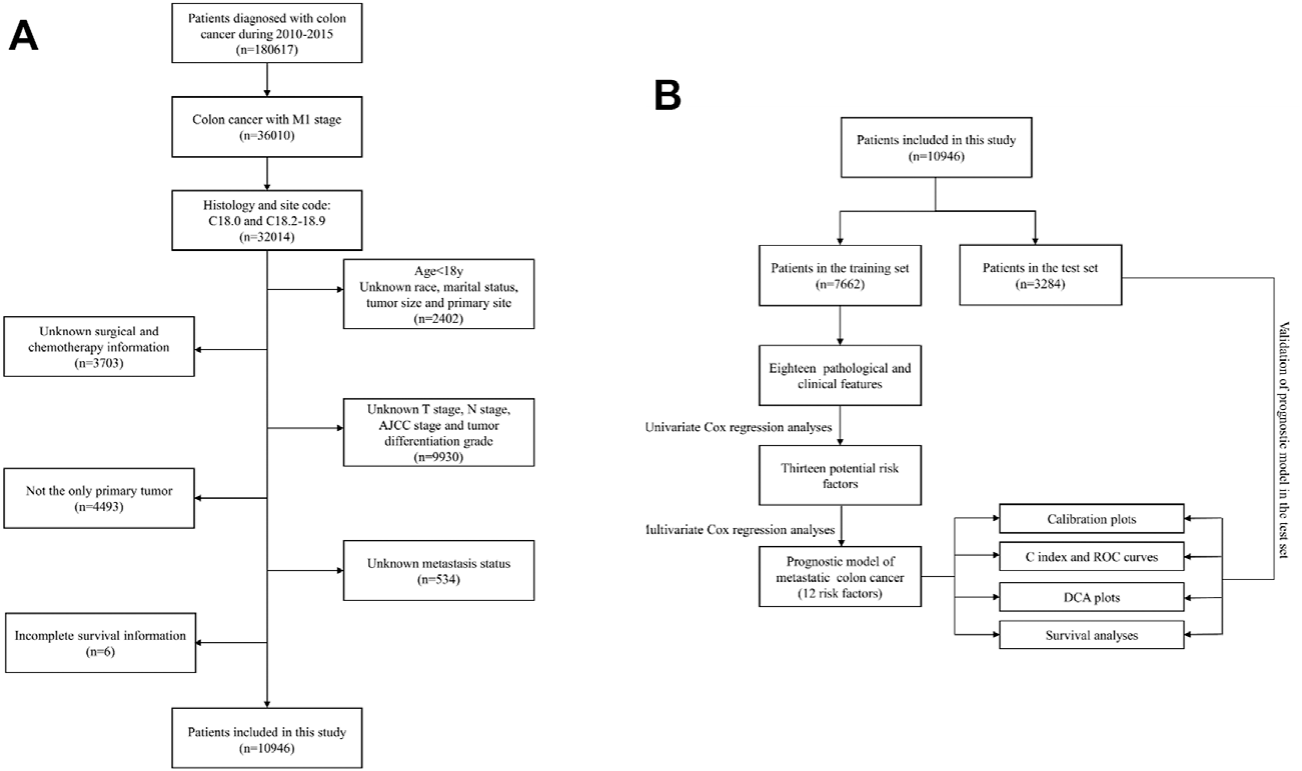
**(A)** Flowchart of the patient screening process. **(B)** Flowchart of the research process of this article.

This study was performed as a retrospective cohort study using the Surveillance, Epidemiology, and End Results (SEER) database (http://seer.cancer.gov/). The SEER database has been updated every other year since 1973 and includes incidence, prevalence, and mortality for a variety of different tumors and can be used to analyze epidemiologic trends in tumors. In this study, data of patients diagnosed with mCC from 2010 to 2015 were retrieved using SEER*Stat version 8.3.6. The selection criteria and screening process are illustrated in Figure 1A.

### Clinical Variables and Outcomes

Clinical variables extracted in this study were as follows: age, race, gender, AJCC stage, T stage, N stage, M stage, treatment method (surgery radiation and chemotherapy), tumor size, marital status, survival status, marital status, tumor primary site, and tumor differentiation grade. The primary outcome was overall survival (OS), which refers to the time from diagnosis until death from any cause or the end of the follow-up period.

### Statistical Analysis

To make the patients in the two groups comparable, after setting the random seed number, 10,946 mCC patients were randomly assigned into two groups by 70 and 30% using R software (Version 4.1.0), one group was used to develop the nomogram (*n* = 7,662), and the other group was used to verify the predictive ability of the constructed model (*n* = 3,284). A flowchart of the research process of this article was presented (Figure 1B).

#### Univariate Cox Analysis and Multivariate Cox Regression for Independent Prognostic Factors

Univariate Cox proportional hazard regression analyses were conducted to detect the predictability of each clinical variable on survival outcomes. These variables comprise tumor characteristics (T-stage, N-stage, M-stage, tumor size), demographic variables (race gender, age) and treatment strategies (surgery, radiation, chemotherapy). Statistically significant factors were then incorporated in the multivariate Cox regression analysis to compute the hazard ratios (HRs) with the corresponding 95% confidence intervals (CIs).

#### Prognostic Nomogram Construction

Since AJCC stage could be inferred from the combination of T-stage N-stage and M-stage, AJCC staging was not included in the Cox regression. Finally, *p* values less than .05 in the multivariate Cox analysis were selected to construct the nomogram via the “survival”, “foreign”, “rms” and “regplot” packages of the R software. Patients were scored on each of the variables in the nomogram and the final multiple scores were summed to give an overall score predicting 1-year, 3-year, and 5-year OS. The median value of the risk score was used as the cut-off value to classify patients into the high-risk group and low-risk group. Kaplan-Meier survival curves were used to fit the correlation between survival time and predicted scores in the high-risk group and low-risk group.

#### Prognostic Nomogram Validation

To determine the validity of the model, an internal dataset was used for validation, which originated from the rest 30% of the SEER database except for the modeling cohort. Discrimination is the ability to differentiate the predictive model from AJCC staging system. It is measured by the area under the curve (AUC) of the ROC curve and the concordance index (C index). The AUC and C-index range from 0.5 to 1, and values above 0.7 represent excellent discrimination. The calibration plot was used to measure how close the predicted risk is to the actual risk. The proximity of the curve to the 45° diagonal demonstrates the strong predictive power of the model. Decision curve analysis (DCA) is an evaluation standard for clinical utility.

#### Dynamic Nomogram Construction

The traditional nomogram is based on different clinical variables to obtain the corresponding scores, and the sum of all scores can be used to predict patient survival at fixed time points such as 1 year, 3 years and 5 years. In this process, it is necessary to make a vertical line to the axis of the total score based on the values of the clinical variables. The process is tedious and the visual observation may have some errors. Therefore, it is of great importance to build an accurate and useful tool. After registering a shinyapps server account and binding it in Rstudio software, the DynNom and PASWR packages were used to compile the multivariable Cox regression results and generate four files: ui.R, server.R, global.R, and functions.R. All files were deployed to the shinyapps server, and then a web-based version of the nomogram was generated.

## Result

### Patient Characteristics

A total of 10,946 patients with mCC were obtained from the SEER database according to inclusion and exclusion criteria. 7,662 of them were divided into the training cohort, 3,284 were in the validation group. In the training set, 3,584 (46.8%) were over 65 years old, 3,911 (51.0%) were male patients, and the majority were white (75.3%), with the remaining being black or other. In terms of the validation cohort, 1,684 (51.3%) patients were male, and 1,577 (48.0%) patients were aged >65 years. Generally, the primary sites of colon cancer were mainly in the right-side colon (58.2%). regarding the treatment strategies, 10,196 (93.1%) patients were treated with surgery and 7,544 (68.9%) patients underwent chemotherapy. A total of 5,619 (51.3%) patients had tumors smaller than 5 cm at diagnosis. Table 1 shows the detailed clinicopathological characteristics of the patients.

**TABLE 1:** Clinicopathological characteristics in mCC patients.

Characteristic	Train cohort (*n* = 7,662)	Test cohort (*n* = 3,284)	*p* value
Age			0.123
<65	4,078 (53.2%)	1,707 (52.0%)	
≥65	3,584 (46.8%)	1,577 (48.0%)	
Race			0.269
White	5,768 (75.3%)	2,471 (75.2%)	
Black	1,191 (15.5%)	532 (16.2%)	
Other	703 (9.2%)	281 (8.6%)	
Sex			0.383
Female	3,751 (49.0%)	1,600 (48.7%)	
Male	3,911 (51.0%)	1,684 (51.3%)	
Primary_site			0.223
Left_side_colon	3,214 (41.9%)	1,351 (41.2%)	
Right_side_colon	4,448 (58.1%)	1,933 (58.8%)	
Grade			0.011
Grade_I	291 (3.80%)	159 (4.84%)	
Grade_II	4,992 (65.2%)	2,099 (63.9%)	
Grade_III	1,915 (25.0%)	854 (26.0%)	
Grade_IV	464 (6.0%)	172 (5.24%)	
Surgery			0.178
Yes	7,125 (93.0%)	3,071 (93.5%)	
No/Unknown	537 (7.0%)	213 (6.5%)	
Radiation			0.318
Yes	311 (4.1%)	127 (3.9%)	
No/Unknown	7,351 (95.9%)	3,157 (96.1%)	
Chemotherapy			0.314
Yes	5,291 (69.1%)	2,253 (68.6%)	
No/Unknown	2,371 (30.9%)	1,031 (31.4%)	
Marital_status			0.334
Married	6,161 (80.4%)	2,651 (80.7%)	
Unmarried	1,501 (19.6%)	633 (19.3%)	
AJCC_stage			0.368
Iva	4,454 (58.1%)	1,900 (57.8%)	
IVb	3,208 (41.9%)	1,384 (42.2%)	
T_stage			0.380
T1+T2+T3	4,294 (56.0%)	1,833 (55.8%)	
T4	3,368 (44.0%)	1,451 (44.2%)	
N_stage			0.198
N0+N1	4,226 (55.2%)	1,782 (54.3%)	
N2	3,436 (44.8%)	1,502 (45.7%)	
M_stage			0.368
M1b	4,454 (58.1%)	1,900 (57.8%)	
M1a	3,208 (41.9%)	1,384 (42.2%)	
Bone_metastasis			0.199
No	7,445 (97.2%)	3,201 (97.4%)	
Yes	217 (2.8%)	83 (2.6%)	
Brain_metastasis			0.116
No	7,584 (99.0%)	3,259 (99.2%)	
Yes	78 (1.0%)	25 (0.8%)	
Liver_metastasis			0.275
No	2,153 (28.1%)	941 (28.6%)	
Yes	5,509 (71.9%)	2,343 (71.4%)	
Lung_metastasis			0.286
No	6,418 (83.8%)	2,765 (84.2%)	
Yes	1,244 (16.2%)	519 (15.8%)	
Tumor_size			0.439
<2 cm	152 (2.0%)	58 (1.8%)	
2–5 cm	3,764 (49.1%)	1,645 (50.1%)	
5–10 cm	3,369 (44.0%)	1,431 (43.5%)	
>10 cm	377 (4.9%)	150 (4.6%)	
Status			0.259
Alive	2,072 (27.0%)	868 (26.5%)	
Dead	5,590 (73.0%)	2,416 (73.5%)	

**TABLE 2:** Univariate and multivariate analysis of training cohort.

Characteristic	Univarite analysis		Multivariate analysis	
	Hazard rate (95%CI)	*p* value	Hazard rate (95%CI)	*p* value
Age				
<65	1		1	
≥65	1.63 (1.55–1.72)	<0.001*	1.36 (1.28–1.43)	<0.001*
Race				
White	1			
Black	1.04 (0.97–1.12)	0.311		
Other	0.92 (0.84–1.01)	0.076		
Sex				
Female	1			
Male	0.98 (0.93–1.03)	0.358		
Primary_site				
Left_side_colon	1		1	
Right_side_colon	1.54 (1.46–1.63)	<0.001*	1.29 (1.22–1.36)	<0.001*
Grade				
Grade_I	1		1	
Grade_II	1.05 (0.91–1.21)	0.523	1.11 (0.97–1.28)	0.13
Grade_III	1.64 (1.42–1.92)	<0.001*	1.54 (1.33–1.78)	<0.001*
Grade_IV	1.69 (1.42–2.12)	<0.001*	1.55 (1.31–1.84)	<0.001*
Surgery				
Yes	1		1	
No/Unknown	1.99 (1.81–2.19)	<0.001*	2.52 (2.28–2.84)	<0.001*
Radiation				
Yes	1			
No/Unknown	1.06 (0.93–1.21)	0.4		
Chemotherapy				
Yes	1		1	
No/Unknown	2.91 (2.76–3.08)	<0.001*	2.96 (2.79–3.13)	<0.001*
Marital_status				
Married	1			
Unmarried	1.06 (0.99–1.13)	0.081		
AJCC_stage				
Iva	1			
IVb	1.56 (1.48–1.64)	<0.001*		
T_stage				
T1+T2+T3	1		1	
T4	1.39 (1.31–1.46)	<0.001*	1.34 (1.27–1.42)	<0.001*
N_stage				
N0+N1	1		1	
N2	1.36 (1.29–1.44)	<0.001*	1.41 (1.34–1.51)	<0.001*
M_stage				
M1b	1		1	
M1a	1.56 (1.48–1.64)	<0.001*	1.38 (1.33–1.46)	<0.001*
Bone_metastasis				
No	1		1	
Yes	1.95 (1.69–2.25)	<0.001*	1.27 (1.12–1.48)	0.0015*
Brain_metastasis				
No	1		1	
Yes	2.04 (1.6–2.59)	<0.001*	1.67 (1.31–2.12)	<0.001*
Liver_metastasis				
No	1		1	
Yes	1.09 (1.02–1.15)	0.006*	1.51 (1.42–1.62)	<0.001*
Lung_metastasis				
No	1		1	
Yes	1.29 (1.2–1.38)	<0.001*	1.14 (1.06–1.23)	<0.001*
Tumor_size				
<2 cm	1		1	
2–5 cm	1.14 (0.94–1.39)	0.183	1.11 (0.91–1.35)	0.31
5–10 cm	1.33 (1.09–1.63)	0.004*	1.18 (0.97–1.44)	0.1
>10 cm	1.63 (1.3–2.04)	<0.001*	1.22 (0.98–1.53)	0.082

Bold values means P value less than 0.05.

### Prognostic Nomogram Construction

**FIGURE 2:**
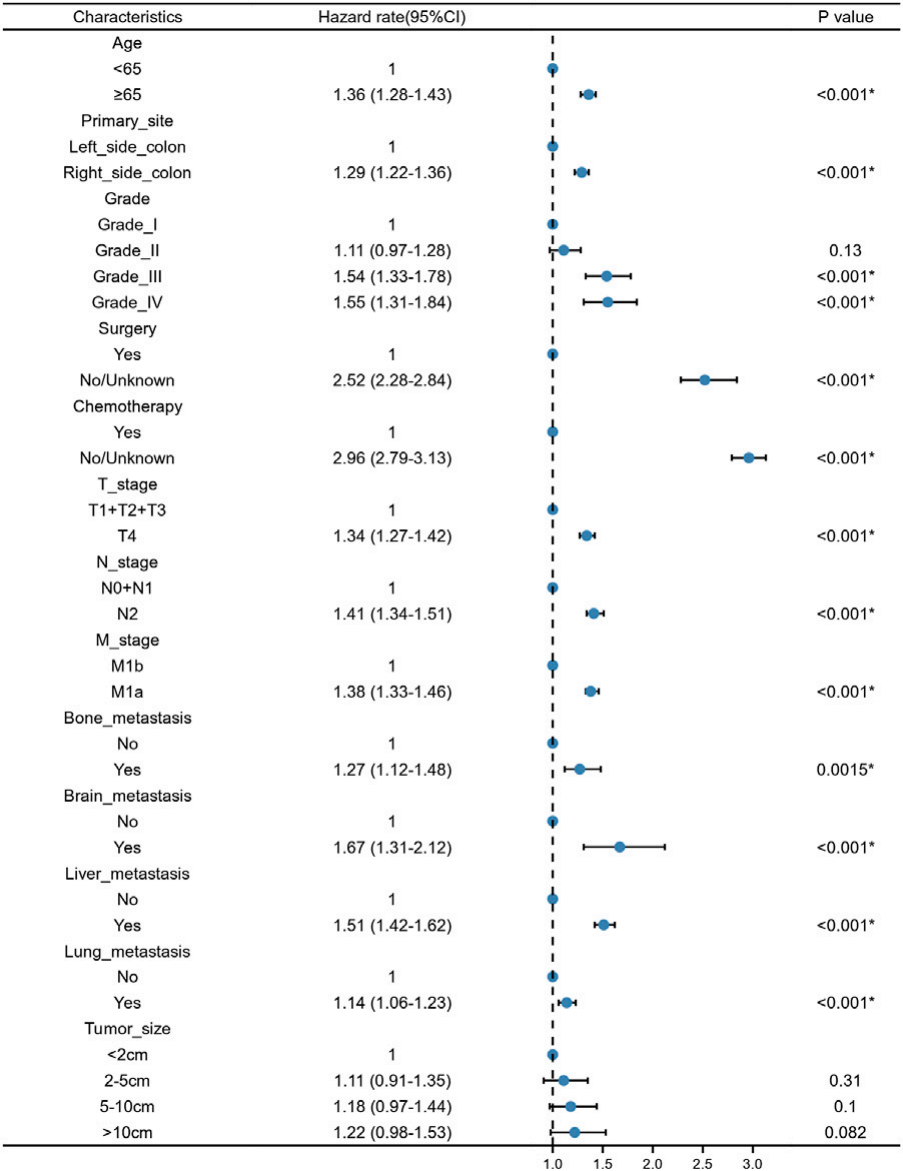
Forest plot for multivariate Cox regression analysis of mCC patients in the training cohort.

**FIGURE 3:**
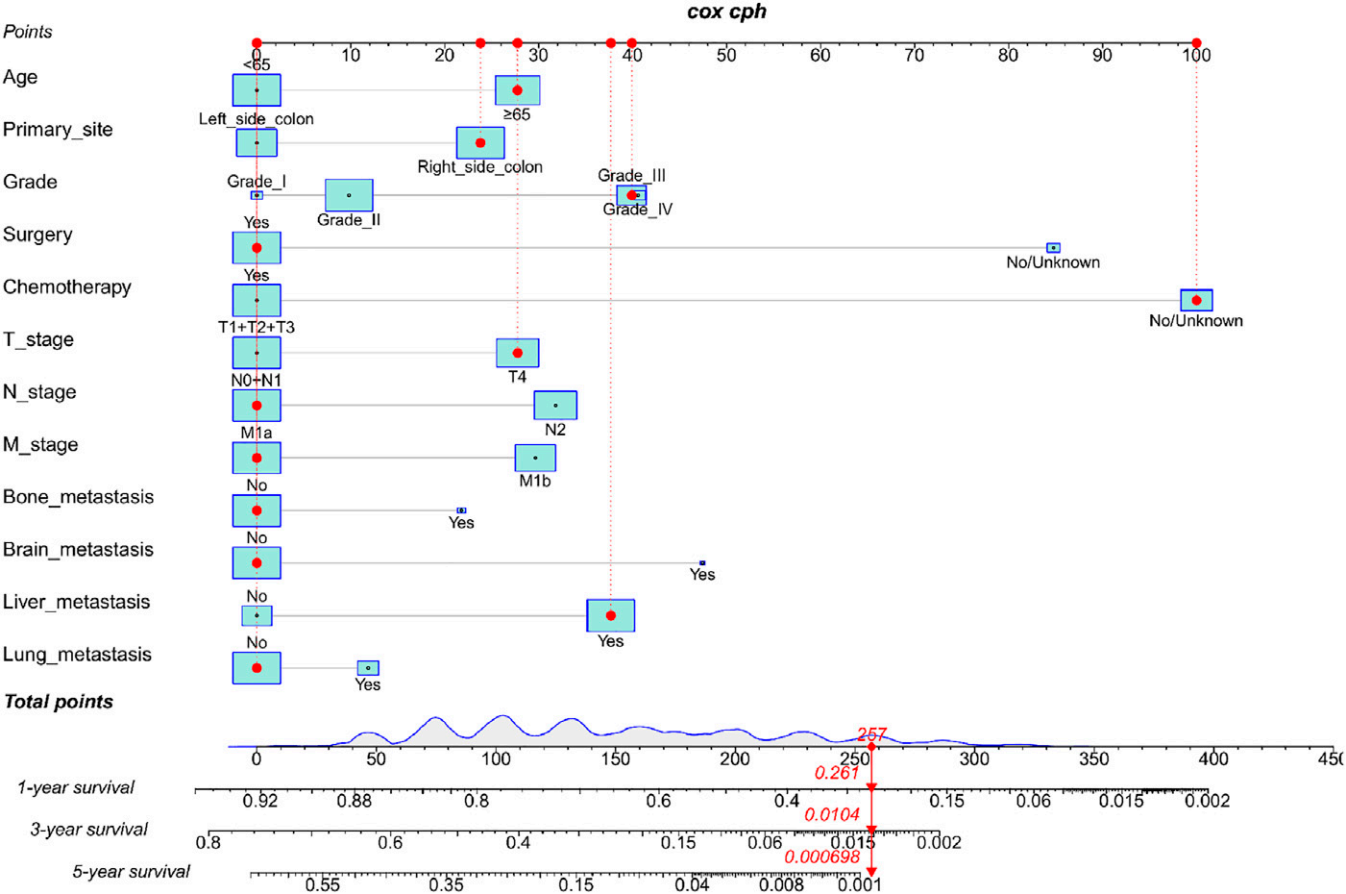
Nomogram for predicting 1-,3-, and 5-year overall survival (OS) for mCC; patients in the training cohort.

We constructed a nomogram using the independent variables identified in the training set that were associated with OS (Figure 2). The potential risk factors were listed as follows: age (<65 years, ≥65 years), primary site (left side colon, right side colon), Grade (Grade I, Grade II, Grade III, Grade IV), surgery (yes, or no/unknown), chemotherapy (yes, or no/unknown), T stage (T1/T2/T3, or T4), N stage (N0/N1, or N2), M stage (M1a, or M1b), bone metastasis (yes, or no), brain metastasis (yes, or no), liver metastasis (yes, or no), and lung metastasis (yes, or no). The nomogram summarizes the scores determined on the scale for each of these risk factors, and by summing the scores for single items in the nomogram, the 1-year, 3-year, and 5-year OS of an individual patient can be predicted based on the total score shown at the bottom of the graph. For instance, a 65-year-old (28 points) patient with Grade-III right-side colon cancer (64 points), with only liver metastasis (38 points) and T4N1M1a (28 points) who received surgery (0 points) and did not undergo chemotherapy (100 points) gets a sum-point of 258, corresponding to predicted 1-, 3-, and 5-year OS of 26.1, 1.04, and 0.07%, respectively (Figure 3). To apply this model for clinical, we add dynamic nomogram. The results of univariate and multivariate Cox regression analyses with HR and 95% CI were listed in Table 2 (https://chenyue123.shinyapps.io/DynNomapp/).

### Nomogram Calibration and Validation

**FIGURE 4:**
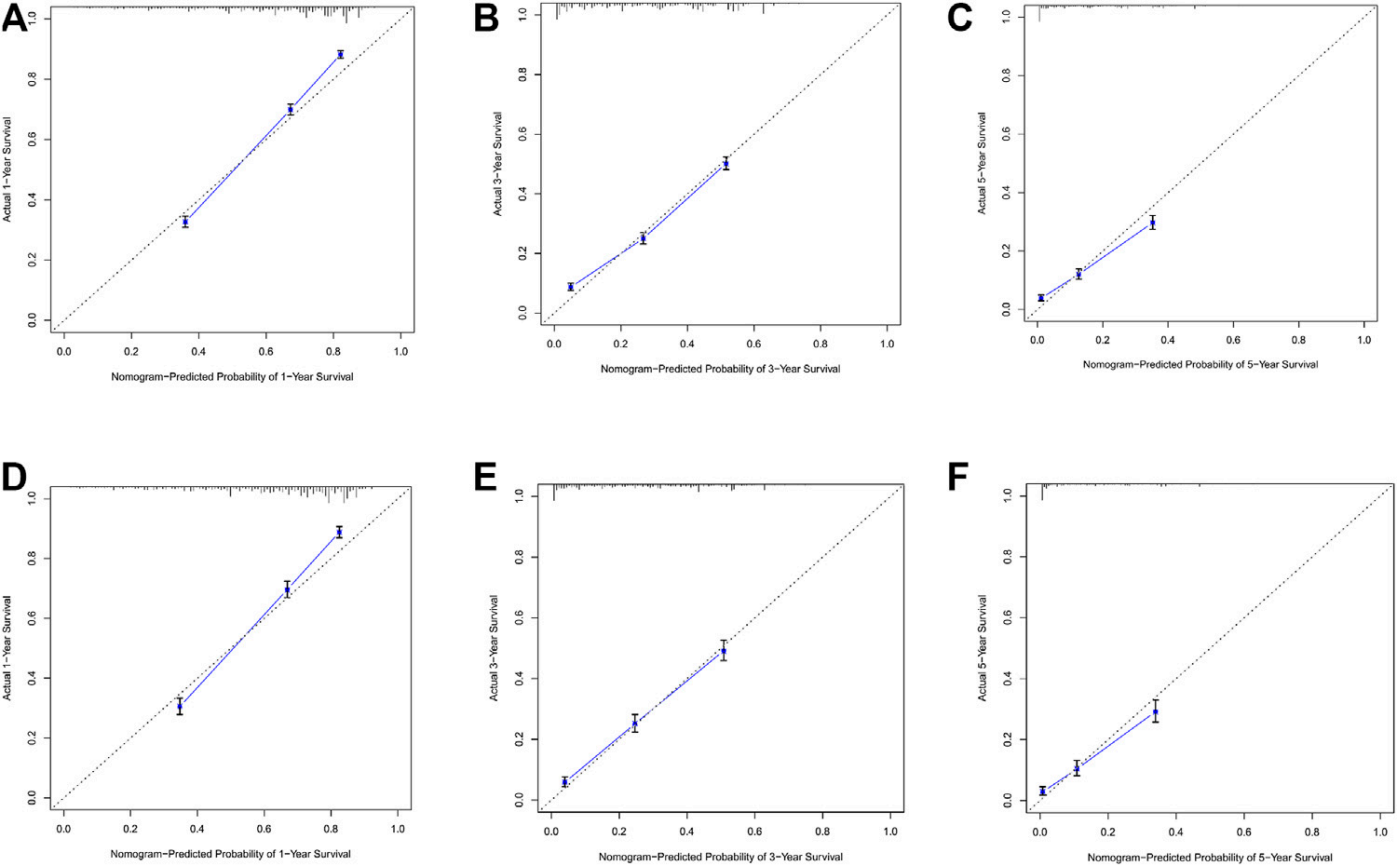
**(A–C)** Nomogram calibration plots to predict 1-,3-, and 5-year overall; survival (OS) in the training cohort; **(D–F)** Nomogram calibration plots to predict 1-,3-, and 5-year overall survival (OS) in the validation cohort.

By observing the calibration plot both in the training cohort and the internal validation cohort can be easily determined that there is a strong agreement between the predicted survival probability and the actual observed results (Figure 4).

**FIGURE 5:**
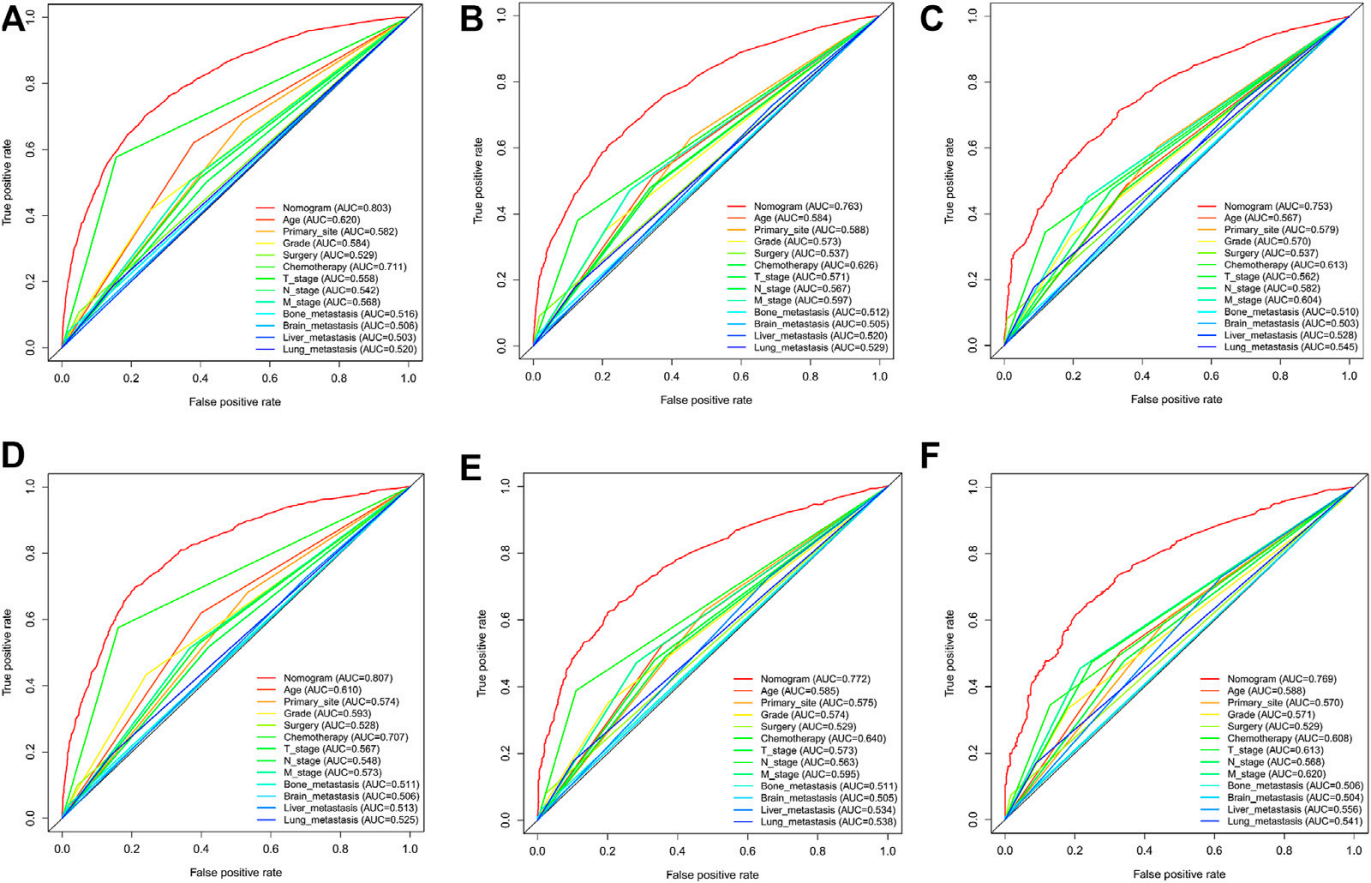
**(A–C)** Nomogram ROC curves to predict 1-,3-, and 5-year overall survival; (OS) in the training cohort; **(D–F)** Nomogram ROC curves to predict 1-,3-, and 5-year; overall survival (OS) in the validation cohort.

**FIGURE 6:**
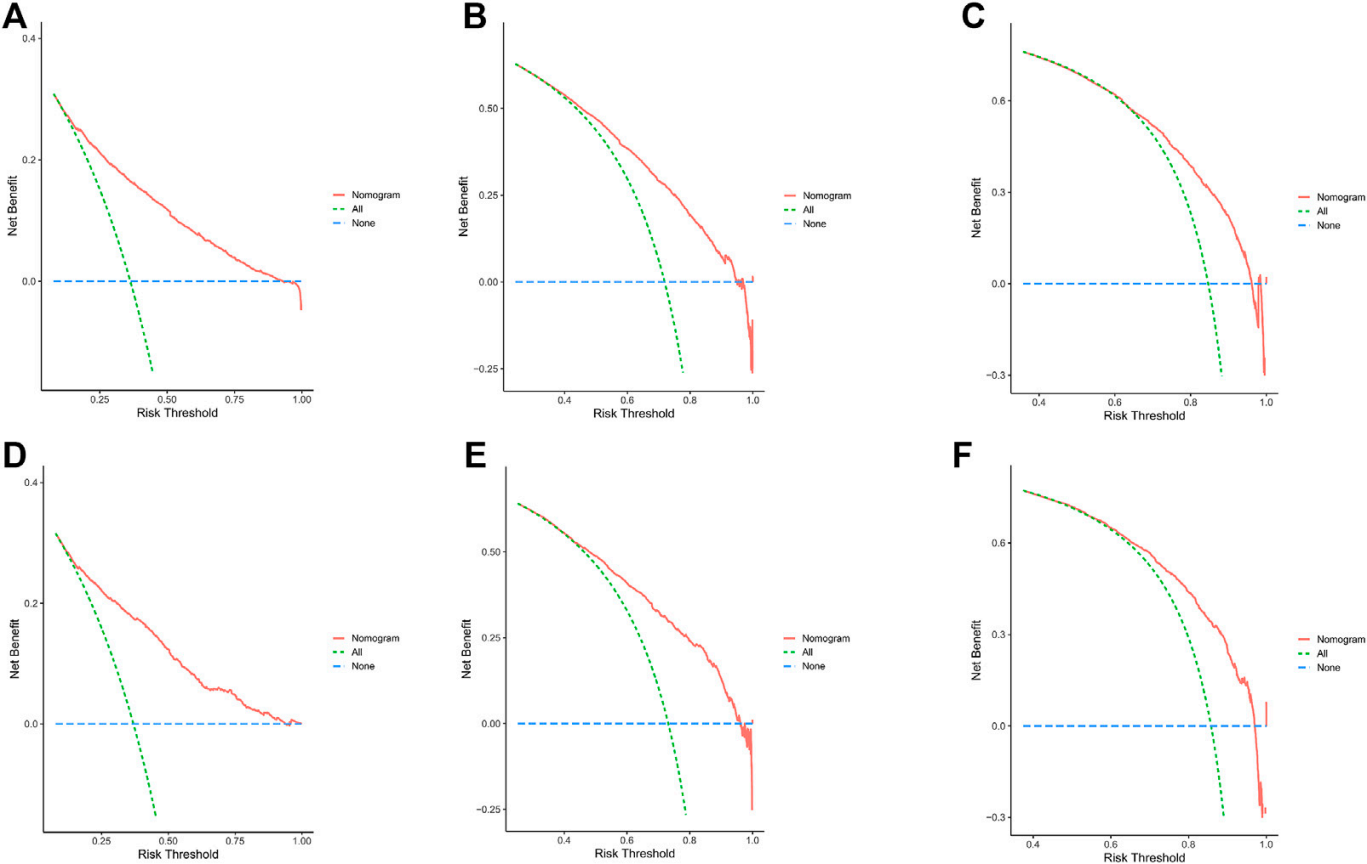
**(A–C)** DCA analysis predicting 1-,3-, and 5-year OS in the training cohort. **(D–F)** DCA analysis predicting 1-,3-, and 5-year OS in the validation cohort.

In the training cohort and internal validation cohort, the C-index values for mCC patients were 0.731 (95% CI 0.725–0.737) and 0.736 (95% CI 0.726–0.746), respectively. Similar to the C-index, we also plotted the ROC curves and calculated the corresponding AUC values. As shown in the ROC plots, the AUC values for each independent prognostic factor were greater than 0.5. By comparing the predictive power of the nomogram with all the independent factors, it was found that the AUC values of the nomogram were higher than each factor at 1, 3, and 5 years. The AUC values of the nomogram predicting OS at 1, 3, and 5 years were 0.803, 0.763, and 0.803 in the training cohort and 0.807, 0.772, and 0.807 in the validation cohort (Figure 5). This result revealed an excellent accuracy of this predictive model. In addition, as presented in Figure 6, the decision curves also indicated better clinical applicability for predicting the overall survival of mCC patients.

Combining the results of DCA curve, C index, ROC curve and calibration curve, we found that the prediction model constructed based on the above-mentioned factors had significant predictive value for OS of mCC patients with high precision and clinical applicability.

### Survival Analyses

**FIGURE 7:**
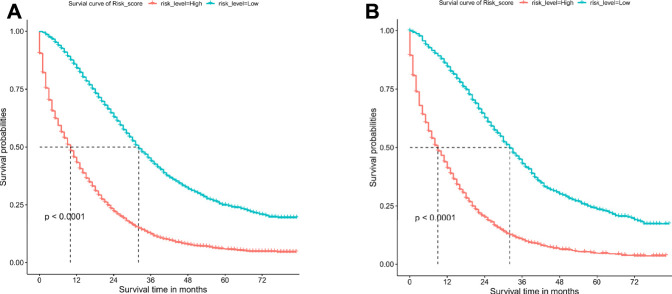
Overall survival (OS) Kaplan-Meier curves for patients in the low- and high-risk groups. **(A)** training cohort. **(B)** internal validation cohort.

The patients were divided into low-risk group and high-risk group by calculating the risk scores based on all independent prognostic factors (median was used as cut-off value). Survival analysis was performed by Kaplan-Meier plots for all independent prognostic factors as well as for the different risk groups (Figure 7, Supplementary Figures S1, S2). It was observed that patients in the low-risk group had a significantly better prognosis than those in the high-risk group (*p* < .001).

### Dynamic Nomogram Construction

To make the constructed nomogram easier to apply in the clinic and more convenient for physicians and patients, we constructed an online version of the nomogram in the form of a web tool (https://chenyue123.shinyapps.io/DynNomapp/). After entering the site, the patient’s clinical variables are shown on the left side of the page and can be entered by selecting the options under each variable. Then, the survival rate of the patients can be calculated.

## Discussion

CC is the fifth leading contributor to cancer-related deaths globally and is cancer that is prone to distant metastases (Sung et al., 2021). Thus, the prognosis of patients with mCC remains a challenging issue for physicians. In Ge’s study, they found thirteen factors associated with colorectal cancer, including age, gender, metastasis status and so on (Ge et al., 2019). In contrast to their study, we developed a model for CC patients only, which is essential to exclude the heterogeneity of colon and rectal cancers. The prognosis of CC patients with liver metastases were established by Zhu and Liu to predict patients’ OS at 1, 2, and 3 years, respectively (Zhu et al., 2020; Liu et al., 2021). Considering that colon cancer does not only metastasize to the liver, but also has a high potential to metastasize to the lung, brain, and bone, a more comprehensive nomogram was developed for patients with mCC. The TNM staging system was recognized as the standard method for staging CC patients (Hari et al., 2013). However, the inherent limitation for the TNM staging system cannot be avoided, as it only emphasizes the primary tumor site, regional lymph node involvement, and distant metastases when assessing patient prognosis, while other factors such as surgery, chemotherapy, and tumor size that affect patient prognosis are not taken into account (Feng et al., 2021; Guevara-Cuellar et al., 2021). Therefore, to assess the survival of mCC patients more comprehensively, nomogram was used to integrate different clinical features to estimate the patient prognosis.

The entire cohort for this study originated from the SEER database, with 70% of the population used to examine the relationship between various potentially influential factors and patient survival outcomes, and the remaining 30% used to assess the predictive power of the models. The newly generated nomograms demonstrated that age, primary site, Grade, surgery, chemotherapy, T stage, N stage, M stage, bone metastasis, brain metastasis, liver metastasis, and lung metastasis could be independent risk factors for mCC. From the perspective of the patient, age and race were investigated to be the important factors influencing the prognosis of mCC patients.

From the perspective of the tumor, T stage, N stage, M stage, brain, lung, bone, and liver metastasis status, tumor primary site and tumor grade can be viewed as predictive factors. The findings of this research are consistent with the previous traditional TNM staging system, which indicate that the prognosis of tumor patients is closely related to primary tumor site, regional lymph node involvement, and distant metastases (Hari et al., 2013). The site of the tumor distant metastasis also matters a lot, compared to those without tumor metastasis, patients with bone metastasis, brain metastasis, lung metastasis, and liver metastasis were at higher risk of death (Chang et al., 2017; Wang et al., 2020; Nakamura et al., 2021). Additionally, our study showed that different tumor sites of colon cancer had significantly different prognoses. The survival prognosis of right side CC is worse than that of left side CC. Such results are consistent with the study of Ge et al. (2019). The reasons for this difference were not clear. However, studies have shown that different sites of colon cancer have different disease biologies, such as microsatellite instability and differences in gene expression (Papagiorgis et al., 2012; Sun, 2021). Tumor histological differentiation grade was also an important factor determining the prognosis of mCC patients. Compared with Grade-I CC patients, the HR for Grade-II, Grade-III and Grade-IV CC patients was 1.03 (0.905–1.16), 1.46 (1.28–1.67), 1.53 (1.31–1.78), respectively. Tumor differentiation grade was based on the degree of interstitial transformation of tumor tissue, including the degree of differentiation, arrangement, the number of nuclear divisions and local infiltration of cancer cells. It can provide a reference basis for clinical treatment and prognosis estimation. Higher grade means the higher malignancy and worse prognosis. From the perspective of the patient’s characteristics, older age was identified as an independent prognostic factor for mCC patients. Many previous studies have addressed the association between age and tumor survival and have concluded that older adults have lower survival rates after developing tumors (Chen et al., 2020; Badic et al., 2021; Pilleron et al., 2021). Also, in Kuai’s study, they constructed a nomogram for liver metastatic CC patients and found age as one of the most important variables (Kuai et al., 2021). A predictive model constructed by Pei et al. for patients with non-metastatic colon cancer also noted the effect of age on patient survival (Pei et al., 2020). From the perspective of treatment, we found that patients would benefit from receiving surgery and chemotherapy. Previous study showed that both primary tumor resection and metastasectomy increased the 5-year OS of mCC from 20 to 50% (Chua and Morris, 2012). The introduction of combination chemotherapy regimens such as bevacizumab and cetuximab have led to a significant improvement in tumor response after chemotherapy in patients with mCC (Niitsu et al., 2015).

This cohort study has some unavoidable limitations. First, as a retrospective study, selection bias might exist in the patient selection process. Moreover, due to the limited clinical information for patients in the SEER database, more valuable clinical factors, such as the specific regimen of radiotherapy, surgical approach, immunotherapy, and intraoperative bleeding, were not considered in the analysis. Last, this study was not externally validated because of the current temporary lack of experimental conditions. To try to make up for this shortcoming, we have allocated the study population in a 7:3 ratio, and 30% of the population was used for internal validation. The results of the internal validation suggest the robustness of the model. Despite these drawbacks, our study has some distinct advantages. First, the SEER database stores information on a large number of CC patients. Therefore, this study contains a sufficient number of samples to make the study results more reliable. Second, to our knowledge, this is the first nomogram to predict the prognosis of mCC patients. Third, this study validated the model in addition to constructing it, and the validation results suggest the stability and reliability of the constructed model. Last but not least, a dynamic nomogram was developed, which was easier to apply and allowed more accurate prediction of patients survival in each month.

## Conclusion

In conclusion, based on the clinical data of mCC patients extracted from the SEER database, we constructed a clinical prediction model with high prognosis prediction accuracy to evaluate the 1-year, 3-year, and 5-year survival of mCC patients. The nomogram developed in this research and other results will help guide clinicians to individualize the diagnosis and treatment for mCC patients.

## Data Availability

The original contributions presented in the study are included in the article/Supplementary Material, further inquiries can be directed to the corresponding authors.

## References

[B1] Badic B. Oguer M. Cariou M. Kermarrec T. Bouzeloc S. Nousbaum J.-B. (2021). Prognostic Factors for Stage III colon Cancer in Patients 80 Years of Age and Older. Int. J. Colorectal Dis. 36 (4), 811–819. 10.1007/s00384-021-03861-6 33528749

[B2] Balachandran V. P. Gonen M. Smith J. J. DeMatteo R. P. (2015). Nomograms in Oncology: More Than Meets the Eye. Lancet Oncol. 16 (4), e173–e180. 10.1016/S1470-2045(14)71116-7 25846097 PMC4465353

[B3] Chang Y.-C. Su C.-Y. Chen M.-H. Chen W.-S. Chen C.-L. Hsiao M. (2017). Secretory RAB GTPase 3C Modulates IL6-STAT3 Pathway to Promote colon Cancer Metastasis and Is Associated with Poor Prognosis. Mol. Cancer 16 (1), 135. 10.1186/s12943-017-0687-7 28784136 PMC5547507

[B4] Chen F. Wang F. Bailey C. E. Murff H. J. Berlin J. D. Shu X. O. (2020). Evaluation of Determinants for Age Disparities in the Survival Improvement of colon Cancer: Results from a Cohort of More Than 486,000 Patients in the United States. Am. J. Cancer Res. 10 (10), 3395–3405. 33163278 PMC7642646

[B5] Chua T. C. Morris D. L. (2012). Therapeutic Potential of Surgery for Metastatic Colorectal Cancer. Scand. J. Gastroenterol. 47 (3), 258–268. 10.3109/00365521.2012.640823 22182312

[B6] Duijster J. Mughini-Gras L. Neefjes J. Franz E. (2021). Occupational Exposure and Risk of colon Cancer: a Nationwide Registry Study with Emphasis on Occupational Exposure to Zoonotic Gastrointestinal Pathogens. BMJ Open 11 (8), e050611. 10.1136/bmjopen-2021-050611 PMC8356182 34376453

[B7] Feng H. Lyu Z. Zheng J. Zheng C. Wu D. q. Liang W. (2021). Association of Tumor Size with Prognosis in colon Cancer: A Surveillance, Epidemiology, and End Results (SEER) Database Analysis. Surgery 169 (5), 1116–1123. 10.1016/j.surg.2020.11.011 33334582

[B8] Ge H. Yan Y. Xie M. Guo L. Tang D. (2019). Construction of a Nomogram to Predict Overall Survival for Patients with M1 Stage of Colorectal Cancer: A Retrospective Cohort Study. Int. J. Surg. 72, 96–101. 10.1016/j.ijsu.2019.10.021 31678689

[B9] Guevara-Cuellar C. A. Soto-Rojas V. E. Echeverry-Molina M. I. Gómez M. Martínez P. (2021). Optimal Allocation of Chemotherapy Schemes for Metastatic Colon Cancer in Colombia. Value Health Reg. Issues 26, 105–112. 10.1016/j.vhri.2021.01.006 34166882

[B10] Hari D. M. Leung A. M. Lee J.-H. Sim M.-S. Vuong B. Chiu C. G. (2013). AJCC Cancer Staging Manual 7th Edition Criteria for colon Cancer: Do the Complex Modifications Improve Prognostic Assessment? J. Am. Coll. Surgeons 217 (2), 181–190. 10.1016/j.jamcollsurg.2013.04.018 PMC4657944 23768788

[B11] Huang Y. Ji L. Zhu J. Mao X. Sheng S. Hao S. (2021). Lymph Node Status and its Impact on the Prognosis of Left‐sided and Right‐sided colon Cancer: A SEER Population‐based Study. Cancer Med. 10, 8708–8719. 10.1002/cam4.4357 34697912 PMC8633222

[B12] Kuai L. Zhang Y. Luo Y. Li W. Li X.-d. Zhang H.-p. (2021). Prognostic Nomogram for Liver Metastatic Colon Cancer Based on Histological Type, Tumor Differentiation, and Tumor Deposit: A TRIPOD Compliant Large-Scale Survival Study. Front. Oncol. 11, 604882. 10.3389/fonc.2021.604882 34712601 PMC8546254

[B13] Lemini R. Attwood K. Almerey T. Gunn J. Yeager T. E. Elias A. W. (2019). Is Metastasectomy a Worthy Option?-The Role of Surgery in Metastatic colon Cancer to Liver and Lungs. J. Gastrointest. Oncol. 10 (6), 1032–1048. 10.21037/jgo.2019.09.06 31949921 PMC6954992

[B14] Liu C. Hu C. Huang J. Xiang K. Li Z. Qu J. (2021). A Prognostic Nomogram of Colon Cancer with Liver Metastasis: A Study of the US SEER Database and a Chinese Cohort. Front. Oncol. 11, 591009. 10.3389/fonc.2021.591009 33738248 PMC7962604

[B15] Nakamura Y. Hokuto D. Koyama F. Matsuo Y. Nomi T. Yoshikawa T. (2021). The Prognosis and Recurrence Pattern of Right- and Left-Sided Colon Cancer in Stage II, Stage III, and Liver Metastasis after Curative Resection. Ann. Coloproctol. 37 (5), 326–336. 10.3393/ac.2020.09.14 32972100 PMC8566149

[B16] Neazy S. A. Mikwar Z. Sameer A. S. Alghamdi K. Alowaydhi H. M. Hashim R. T. (2021). Risk Factors, Clinical Manifestations and Treatment Outcomes of Colon Cancer Patients in National Guard Hospital in Jeddah, Saudi Arabia. Cureus 13 (9), e18150. 10.7759/cureus.18150 34703688 PMC8529408

[B17] Niitsu H. Hinoi T. Shimomura M. Egi H. Hattori M. Ishizaki Y. (2015). Up-front Systemic Chemotherapy Is a Feasible Option Compared to Primary Tumor Resection Followed by Chemotherapy for Colorectal Cancer with Unresectable Synchronous Metastases. World J. Surg. Onc 13, 162. 10.1186/s12957-015-0570-1 PMC4426172 25908502

[B18] Papagiorgis P. C. Zizi A. E. Tseleni S. Oikonomakis I. N. Nikiteas N. I. (2012). The Pattern of Epidermal Growth Factor Receptor Variation with Disease Progression and Aggressiveness in Colorectal Cancer Depends on Tumor Location. Oncol. Lett. 3 (5), 1129–1135. 10.3892/ol.2012.621 22783405 PMC3389629

[B19] Pei J.-P. Zhang C.-D. Liang Y. Zhang C. Wu K.-Z. Zhao Z.-M. (2020). Novel Nomograms Individually Predicting Overall Survival of Non-metastatic Colon Cancer Patients. Front. Oncol. 10, 733. 10.3389/fonc.2020.00733 32435623 PMC7218119

[B20] Pilleron S. Maringe C. Charvat H. Atkinson J. Morris E. J. A. Sarfati D. (2021). The Impact of Timely Cancer Diagnosis on Age Disparities in colon Cancer Survival. J. Geriatr. Oncol. 12 (7), 1044–1051. 10.1016/j.jgo.2021.04.003 33863698

[B21] Skarkova V. Kralova V. Vitovcova B. Rudolf E. (2019). Selected Aspects of Chemoresistance Mechanisms in Colorectal Carcinoma-A Focus on Epithelial-To-Mesenchymal Transition, Autophagy, and Apoptosis. Cells 8, 234. 10.3390/cells8030234 PMC6468859 30871055

[B22] Sucandy I. Giovannetti A. Spence J. Ross S. Rosemurgy A. (2020). Robotic Partial Left Hepatectomy for Metastatic Colon Cancer to the Liver. Application of Minimally Invasive Technique in Cancer Surgery. The Am. Surgeon, 000313482095287. 10.1177/0003134820952873 33345587

[B23] Sun B. L. (2021). Current Microsatellite Instability Testing in Management of Colorectal Cancer. Clin. Colorectal Cancer 20 (1), e12–e20. 10.1016/j.clcc.2020.08.001 32888812

[B24] Sung H. Ferlay J. Siegel R. L. Laversanne M. Soerjomataram I. Jemal A. (2021). Global Cancer Statistics 2020: GLOBOCAN Estimates of Incidence and Mortality Worldwide for 36 Cancers in 185 Countries. CA A. Cancer J. Clin. 71 (3), 209–249. 10.3322/caac.21660 33538338

[B25] Valentini V. van Stiphout R. G. P. M. Lammering G. Gambacorta M. A. Barba M. C. Bebenek M. (2011). Nomograms for Predicting Local Recurrence, Distant Metastases, and Overall Survival for Patients with Locally Advanced Rectal Cancer on the Basis of European Randomized Clinical Trials. Jco 29 (23), 3163–3172. 10.1200/JCO.2010.33.1595 21747092

[B26] van der Stok E. P. Spaander M. C. W. Grünhagen D. J. Verhoef C. Kuipers E. J. (2017). Surveillance after Curative Treatment for Colorectal Cancer. Nat. Rev. Clin. Oncol. 14, 297–315. 10.1038/nrclinonc.2016.199 27995949

[B27] Wang J. Li S. Liu Y. Zhang C. Li H. Lai B. (2020). Metastatic Patterns and Survival Outcomes in Patients with Stage IV colon Cancer: a Population-Based Analysis. Cancer Med. 9, 361–373. 10.1002/cam4.2673 31693304 PMC6943094

[B28] Wang Y. Liu J. Ren F. Chu Y. Cui B. (2021). Identification and Validation of a Four-Long Non-coding RNA Signature Associated with Immune Infiltration and Prognosis in Colon Cancer. Front. Genet. 12, 671128. 10.3389/fgene.2021.671128 34290738 PMC8287327

[B29] Zhu Y.-J. Chen Y. Hu H.-Y. Zhou Y.-W. Zhu Y.-T. Liu J.-Y. (2020). Predictive Risk Factors and Online Nomograms for Synchronous Colon Cancer with Liver Metastasis. Front. Oncol. 10, 1681. 10.3389/fonc.2020.01681 33123459 PMC7566411

