# Supplementary figures and images for: Survival Nomogram for Metastasis Colon Cancer Patients Based on SEER Database
Source: Front Genet. 2022 Feb 9;13:832060. doi: 10.3389/fgene.2022.832060 (PMC8864078; doi:10.3389/fgene.2022.832060)

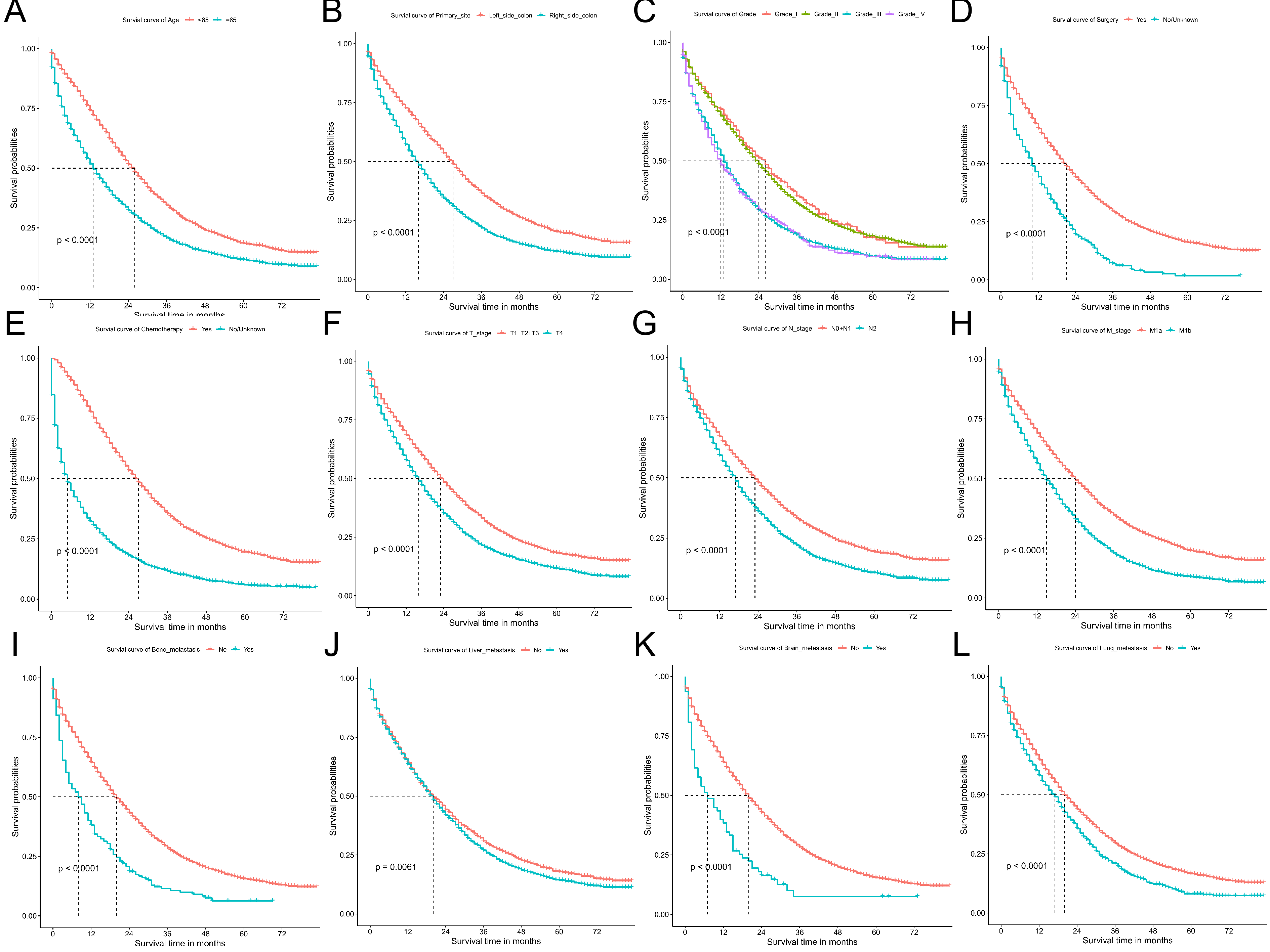

Supplement: Supplementary file 1 [file Image1.TIFF]

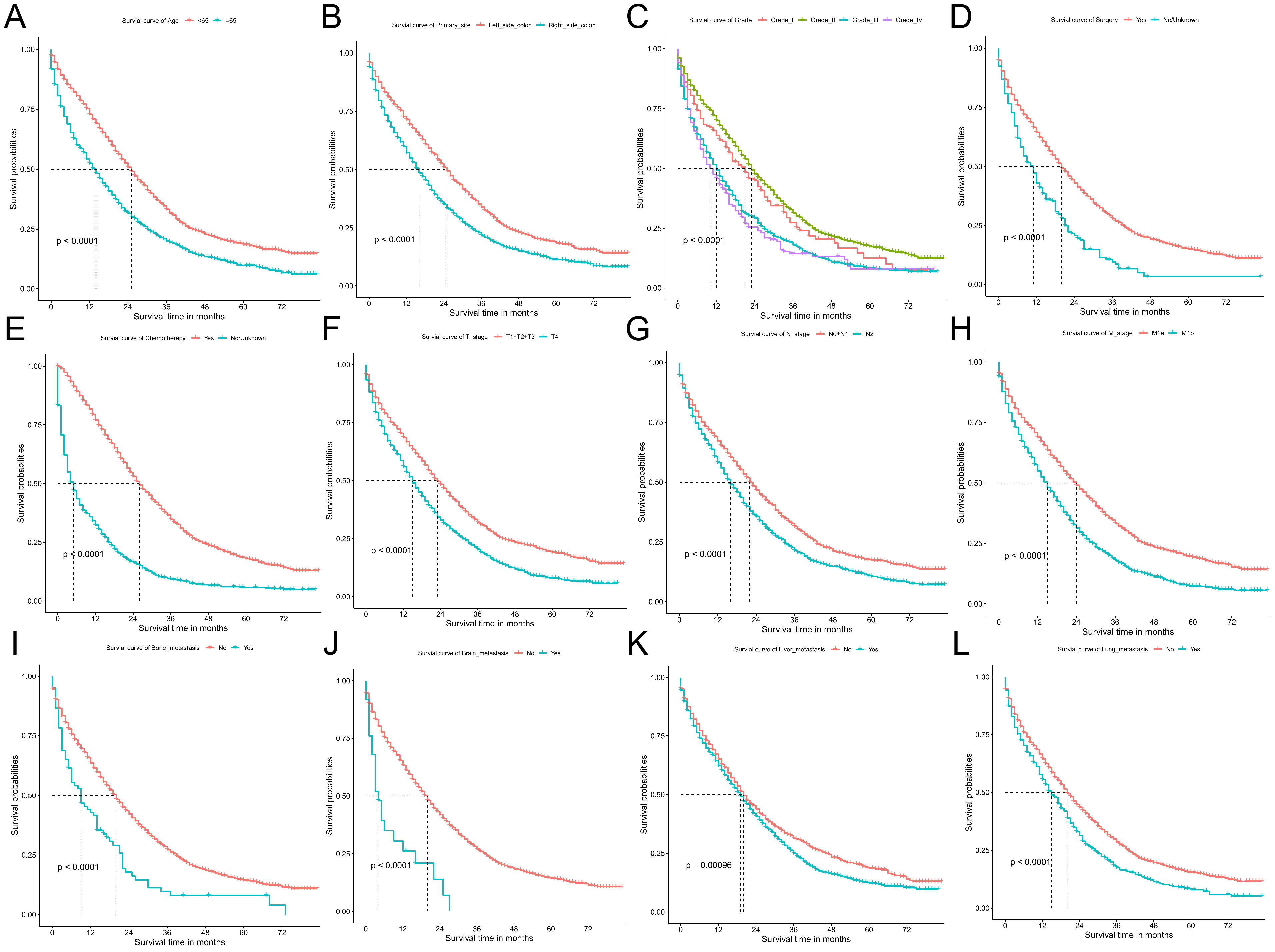

Supplement: Supplementary file 2 [file Image2.TIFF]
